# Using herbarium collections to study genetic responses to global change

**DOI:** 10.1111/nph.70454

**Published:** 2025-08-08

**Authors:** Lucas Eckert, Isaac Eckert, Olivia Rahn, Cameron P. So, Rowan D. H. Barrett

**Affiliations:** ^1^ Department of Biology McGill University Montreal QC H3A 1B1 Canada; ^2^ Quebec Centre for Biodiversity Science Montreal QC H3A 1B1 Canada

**Keywords:** adaptation, climate change, extinction, genetics, global change, herbarium collections

## Abstract

Earth's *c*. 406 million herbarium specimens represent a largely untapped resource of genetic data that could transform our understanding of global plant populations. Advances in DNA sequencing have made the extraction of genetic data from these preserved specimens increasingly feasible, enabling new insights into plant biodiversity and evolutionary dynamics. However, researchers have only begun to leverage these historical genomes, and the vast majority of this resource remains unexplored. In this viewpoint, we discuss how herbarium collections can be used to study the genetic responses of plant populations to global change. Several promising areas of research include using herbaria for genetic monitoring, studying local extinction dynamics, identifying targets of selection under environmental change, and validating genomic predictions through hindcasting. Herbarium collections represent a unique and underutilized resource, the mobilization of which has the potential to enhance our understanding of plant responses to global change and inform conservation efforts.

## Herbarium collections as treasure troves of genetic data

Over the past half‐millennium, humans have collected, preserved, and stored over 406 million plant specimens that, together with other natural history collections, represent the largest biodiversity dataset on Earth (Page *et al*., 2015). Increasingly, researchers are turning towards these historical collections as a source of irreplicable biodiversity data (Meineke *et al*., 2018): including historical species occurrence records (Calinger, 2015), functional traits (Heberling, 2022), biotic interactions (Meineke & Davies, 2018), and genomes (Pont *et al*., 2019). As accessibility to these collections has grown, so too has their utility in studying how biodiversity is responding to global change (Meineke *et al*., 2018; Lang *et al*., 2019). One promising area of research focuses on leveraging the genomes housed in herbarium collections (Bieker & Martin, 2018; Burbano & Gutaker, 2023; Davis & Knapp, 2025) to study genetic responses to global change: however, relatively few studies have explored their potential applications in this domain, leaving their ability to supply genetic data largely unrealized.

Historically, the challenge of generating genetic data from herbarium specimens was in DNA extraction and sequencing, as the DNA can be highly degraded and fragmented (Staats *et al*., 2011). However, recent advances in technologies and workflows have made this process more feasible, more effective, and less expensive (Gutaker & Burbano, 2017; Bieker & Martin, 2018; Kistler *et al*., 2020). While the extraction of DNA from herbarium specimens was possible decades ago, these advances have enabled genetic analysis of greater numbers of specimens across deeper time scales and with a diversity of sequencing approaches, including whole‐genome sequencing (Staats *et al*., 2013; Olofsson *et al*., 2016; Exposito‐Alonso *et al*., 2018), targeted sequencing (Hart *et al*., 2016; Sánchez Barreiro *et al*., 2017; Lang *et al*., 2020), and SNP genotyping (Vandepitte *et al*., 2014; Nygaard *et al*., 2022).

An additional challenge in working with genetic data from herbarium collections lies in aggregating individual specimens into study designs suitable for testing eco‐evolutionary hypotheses. Traditionally, herbarium collections were primarily collected for taxonomic inventories and species descriptions (Heberling & Isaac, 2017), meaning collections of many individuals of the same species from the same site at the same time are relatively uncommon; unfortunately, this is exactly the sampling effort required for most population genetic analyses. However, several recent studies have overcome this challenge by clustering specimens together in space and time to represent historical populations, often aggregating specimens from multiple institutions (e.g. Nygaard *et al*., 2022; Viveiros‐Moniz *et al*., 2025). Given the sheer size of herbarium collections, it is likely this approach would be effective for many species, particularly in well‐sampled regions, yet the extent to which this is possible remains unclear. Promisingly, the ongoing digitization of herbarium collections will enhance our understanding of the temporal and spatial distribution of specimens, making this approach increasingly feasible.

To guide future research in light of these advancements, we discuss several areas of research for which herbarium specimens can offer unique insights, including genetic monitoring, studying local extinction dynamics, identifying targets of selection, and validating genomic predictions. Novel insights in these areas could transform our knowledge of how plant populations are coping with global change and help inform conservation measures and policy decisions.

## Impacts of global change on genetic variation

Climate change, habitat fragmentation, pollution, and other human impacts on the environment are expected to have significant effects on population‐level genetic variation (Aitken *et al*., 2008) and recent studies suggest these effects are widespread (Shaw *et al*., 2025). Due to the relationship between metrics of genetic variation and population health (Booy *et al*., 2000; Reed & Frankham, 2003), monitoring genetic change is increasingly considered central to biodiversity monitoring and conservation programs (DeWoody *et al*., 2021; Convention on Biological Diversity, 2022). While many metrics can be used to measure changes in genetic variation, four variables have been proposed as genetic Essential Biodiversity Variables: genetic diversity, genetic differentiation, inbreeding, and effective population size (Hoban *et al*., 2022). We generally expect that all these metrics can be affected by human impacts on the environment. For instance, habitat loss and environmental change can cause population declines, lowering effective population sizes and genetic diversity, and increasing the level of inbreeding (Pauls *et al*., 2013). Habitat fragmentation can exacerbate the problem, decreasing gene flow between populations (increasing genetic differentiation), further reducing genetic diversity and increasing the level of inbreeding (Young *et al*., 1996). While there is mounting evidence of anthropogenic impacts on population‐level genetic variation (Vranckx *et al*., 2012; González *et al*., 2020), available data often lack the temporal scale required to confidently detect and attribute genetic change. More fundamentally, genetic data only exist for a small fraction of species, with severe taxonomic and spatial biases in our knowledge (Leigh *et al*., 2021). To understand the genetic changes that are occurring in natural populations, we need more genetic data across greater taxonomic, spatial, and temporal scales – to this end, herbaria and their collections could offer (part of) the solution.

### Measuring changes in metrics of genetic variation

Herbarium specimens offer a valuable opportunity to broaden the temporal scale of genetic data. This is particularly important for studies of global change as we often lack historical baselines that predate contemporary climate change and other human impacts (Lang *et al*., 2019). Quantifying genetic diversity and related metrics from herbarium specimens can provide those historical baselines and allow us to directly measure temporal change (Díez‐del‐Molino *et al*., 2018). This is directly relevant to conservation as detecting changes in genetic metrics is more informative than merely quantifying contemporary values; for example, a population that has seen recent declines is likely at greater risk than one that has remained small for many generations (Kohn *et al*., 2006).

**Fig. 1:**
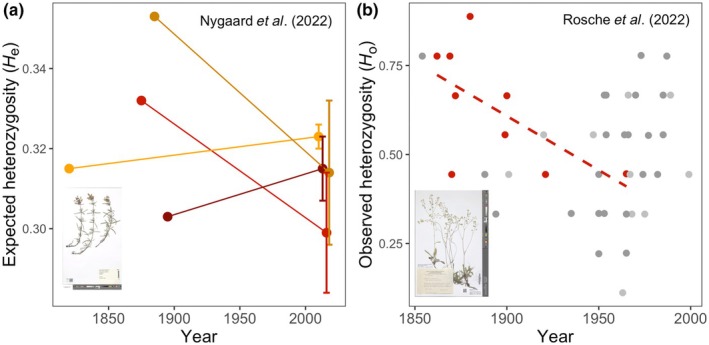
Using herbarium specimens to estimate historical metrics of genetic variation. (a) Nygaard *et al*. (2022) aggregated specimens into both historical and modern populations in multiple locations (different colors) and estimated expected heterozygosity. Note that some populations were excluded from the original dataset for clarity. (b) Rosche *et al*. (2022) regressed observed heterozygosity of individual specimens against time in both extant (light and dark grey) and extinct (red) populations.

Viveiros‐Moniz *et al*. (2025) propose two approaches to using herbarium specimens to measure changes in genetic diversity. The first approach aggregates specimens from the same location and time point into historical populations. When specimens do not cluster neatly into discrete populations, the second approach regresses individual‐level metrics of genetic variation (e.g. observed heterozygosity) against time and other relevant predictors (e.g. altitude; see Fig. 1b). While the latter approach may be more widely applicable due to more lenient sample requirements, the former allows for more accurate estimation of a greater range of metrics as well as the estimation of confidence intervals (e.g. Fig. 1a) and thus is preferable whenever possible.

A few studies have already used herbarium specimens to estimate historical metrics of genetic variation and temporal changes. For example, Nygaard *et al*. (2022) implement both of the approaches described above, with genotyping data from herbarium specimens dating back to 1820 to track temporal changes in genetic diversity and differentiation in northern dragonhead (*Dracocephalum ruyschiana*), a species experiencing severe population declines in Norway. By aggregating specimens into historical populations, they show variable temporal trends in expected heterozygosity across populations, with no clear global trend (Fig. 1a). Studies like that of Nygaard *et al*. demonstrate the utility of herbarium specimens for investigating genetic change over long time scales. Choice taxa to prioritize may include ecologically significant species, species that sustain human livelihoods (Pironon *et al*., 2024; Obiar *et al*., 2025), or those that are imperilled or predicted to be threatened but lack proper assessment (Bachman *et al*., 2024).

### Genetic signals of extinction

A unique application of genetic data from historical collections is the potential to quantify changes in genetic variation preceding local extinction (Albani Rocchetti *et al*., 2021). For example, recent work by Rosche *et al*. (2022) used herbarium specimens to unveil population‐level genetic signatures of local extinction in a subspecies of *Biscutella laevigata*, a flowering perennial herb found across much of central Europe. Using 81 herbarium specimens from both extant and extinct populations (the status of which they confirmed by revisiting collection sites), they were able to quantify the genetic trends of populations through time to assess which of these trends led to extinction events and estimate the consequences of those extinction events on species‐wide genetic diversity. Their analysis revealed that while past extinction events did not impact species‐wide mean genetic diversity, they did lead to the irreversible loss of specific genetic clusters. In one population, they observed a significant decrease in Observed Heterozygosity (*H*_o_) over time preceding extinction, and no significant trend in *H*_o_ in the populations that persisted (Fig. 1b), suggesting that this metric can indeed be indicative of population decline and impending extinction.

This study by Rosche *et al*. (2022) is one of the first of its kind to track the population genetic signatures that precede local extinction and potentially indicate or contribute to extinction risk. Promisingly, their methodology of identifying populations in the herbarium record and using field surveys to determine contemporary persistence or population size should be widely applicable. This is of increasing importance as metrics like genetic diversity and effective population size are beginning to be adopted into conservation policy and decision making, with the intent of predicting and tracking extinction risk (Convention on Biological Diversity, 2022). However, the relationship between these commonly used genetic metrics and extinction risk seems to be weakly predictive and highly variable across taxa (Teixeira & Huber, 2021; Schmidt *et al*., 2023). Recently, more nuanced metrics have been proposed to more accurately assess and predict population genomic health (Bosse & van Loon, 2022; Chung *et al*., 2023). For example, quantifying runs of homozygosity (ROHs) might be a better indicator of the cost of inbreeding depression than simple inbreeding coefficients (Kardos *et al*., 2016; Ceballos *et al*., 2018). Similarly, quantifying the number of deleterious mutations (mutational load) might better capture the mechanisms that plague small populations compared to assessing population size alone (Bosse & van Loon, 2022; Dussex *et al*., 2023). However, the association between extinction risk and even these more tailored metrics remains largely up for debate (van der Valk *et al*., 2019; Grossen *et al*., 2020; von Seth *et al*., 2021). Herbarium specimens could offer much needed empirical tests of whether changes in genetic metrics accurately predict local extinction. These types of studies would also permit testing whether particular alleles are associated with population extinction or persistence, potentially uncovering alleles beneficial to coping with global change. As our understanding of the genetic signatures of extinction grows, genetic monitoring programs will benefit from broader knowledge of the genetic processes and indicators that foreshadow impending extinctions and subsequent biodiversity loss.

## Adaptive responses to global change

Humans are imposing myriad selective pressures upon plant populations (Jump & Peñuelas, 2005; Parmesan, 2006). Beyond exploring how these human impacts might alter metrics of genetic variation, herbarium specimens also offer the opportunity to study putative adaptive responses to global change. Indeed, herbarium specimens are already being widely used to document phenotypic changes in plant populations in response to these selective pressures. For example, specimens have been used to document changes in plant phenology (Primack *et al*., 2004; Panchen *et al*., 2012; Everill *et al*., 2014; Davis *et al*., 2015), morphology (Guerin *et al*., 2012; Leger, 2013), and physiology (McLauchlan *et al*., 2010; Bonal *et al*., 2011; DeLeo *et al*., 2020) in response to climate change. With the increasing feasibility of obtaining high‐quality genetic data from herbarium specimens, these collections also offer the opportunity to study adaptive responses at the genetic level, thereby making it possible to distinguish between genetic evolutionary change and plastic phenotypic responses (Parmesan, 2006). Additionally, exploring signatures of selection at the genetic level can elucidate selection on phenotypes that are not commonly measured, potentially uncovering selection on previously neglected traits. Finally, while the genetic basis of phenotypic traits can be studied using contemporary specimens, the genes that contribute to contemporary variation may not be the same genes that have experienced historical selection and contributed to historical changes (Shaw, 2019), providing another reason to quantify historical changes at the genetic level.

### Identifying signatures of selection

**Fig. 2:**
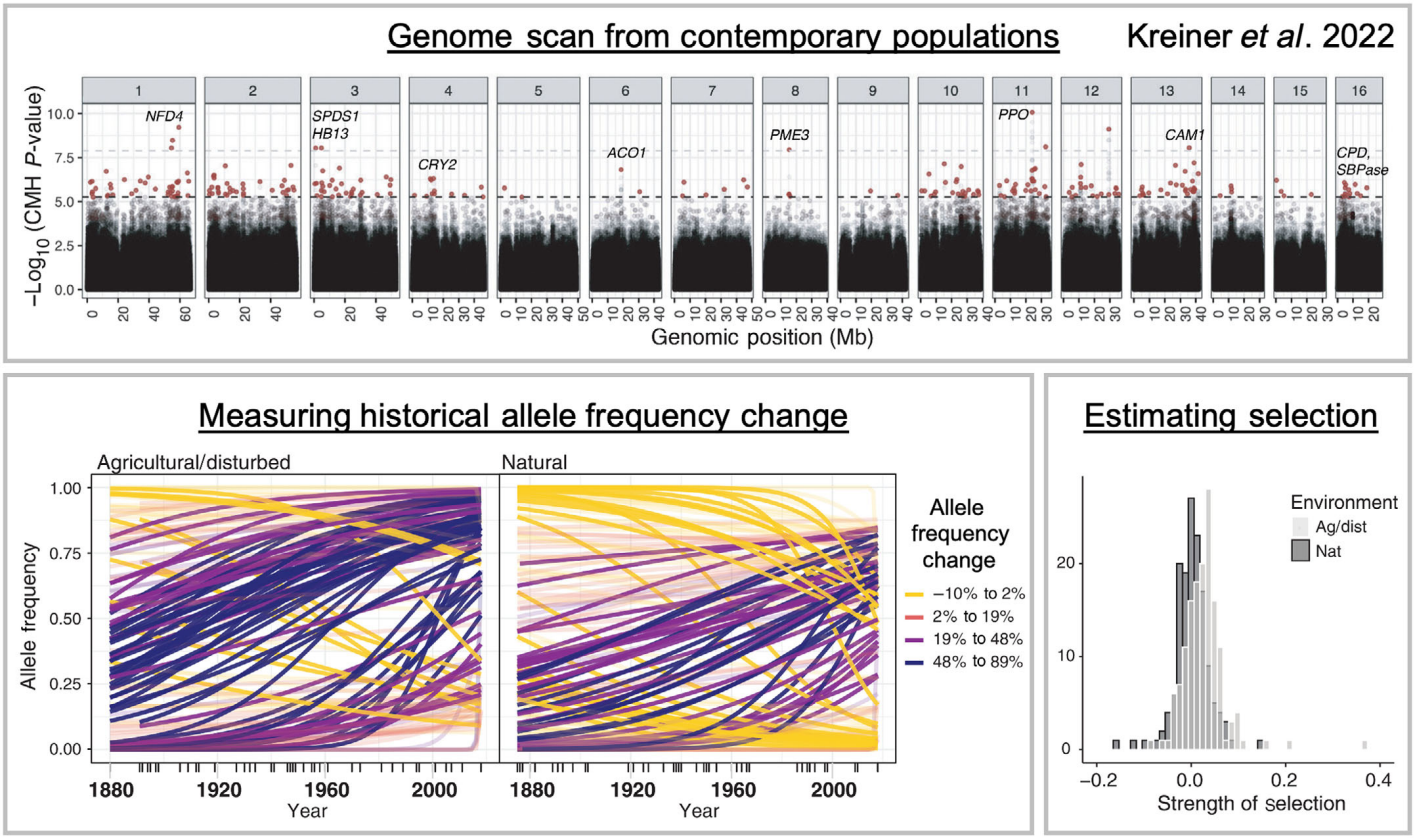
Measuring historical selection, adapted from Kreiner *et al*. (2022). Kreiner *et al*. contrasted contemporary populations of *Amaranthus tuberculatus* in agricultural vs natural habitats to identify alleles associated with agricultural habitats via a genome scan. Using herbarium specimens, they tracked historical allele frequency changes at these loci in both habitat types, finding that they increased significantly and rapidly in agricultural habitats, with slower changes in natural habitats (likely driven by gene flow rather than selection). They estimated the strength of selection on these alleles in both habitats to confirm that they are under positive selection in agricultural habitats.

The field of evolutionary genetics has developed many methods for identifying signatures of selection from genetic data, and several studies have already deployed these methods with data from herbarium specimens to document the genetic basis of adaptation to environmental change. For example, Lang *et al*. (2024) tracked genotypic changes at 24 genes linked to stomatal development in *Arabidopsis thaliana* to show that the commonly observed decrease in stomatal density due to climate change does indeed have a significant genetic basis (rather than being purely plastic). Kreiner *et al*. (2022) contrasted contemporary populations of *Amaranthus tuberculatus* in agricultural vs natural habitats to identify genes associated with adaptation to agricultural land use and then quantified historical selection on these genes using herbarium specimens (Fig. 2). Similarly, Vandepitte *et al*. (2014) contrasted contemporary populations between the native and invasive ranges of *Sisymbrium austriacum* (subsp. *chrysanthum*) and genotyped herbarium specimens to identify selection on genes related to flowering time during the early phases of range expansion. A few studies have even directly contrasted historical and contemporary samples to identify targets of selection. For instance, Bieker *et al*. (2022) used this method to find genes under selection during range expansion in the invasive *Ambrosia artemisiifolia*. Similarly, Gutaker *et al*. (2019) sequenced historical potato (*Solanum tuberosum*) specimens to identify alleles adapted to longer days and shorter growing seasons following introduction to Europe.

The methods used in these studies can be broadly applied to identify adaptive responses to global change. While contrasts between historical and contemporary populations (as in Gutaker *et al*., 2019; Bieker *et al*., 2022) offer the most direct method of identifying targets of selection (similar to evolve and resequence experiments; Long *et al*., 2015), this is best suited to whole‐genome (or otherwise very dense) sequence data, which can be challenging to generate from historical specimens. Alternatively, researchers can use appropriate contrasts between contemporary populations to identify putative targets of selection and then test for change in allele frequencies at these specific genomic regions in herbarium specimens (Kreiner *et al*., 2022; Fig. 2). Finally, researchers can exploit our growing understanding of the genetic basis of plant phenotypes to select genes or gene families to target. Identifying the genes that contribute to adaptation is a major goal in evolutionary biology (Stinchcombe & Hoekstra, 2008; Bomblies & Peichel, 2022; Lasky *et al*., 2023) and is paramount in understanding the past, current, and future adaptive potential of plant populations (Anderson *et al*., 2011; Anderson & Song, 2020).

### Historically validating genomic predictions

In addition to studying historical adaptation to global change, herbarium collections also offer an opportunity to validate our predictions of future adaptive responses. Increasingly, genotype‐environment associations (GEAs) are being used to predict adaptation or maladaptation to future climatic conditions (Capblancq *et al*., 2020a). In this field, researchers identify genes or gene families putatively involved in local adaptation to climate by correlating allele frequencies with contemporary climatic variables (Coop *et al*., 2010; Hoban *et al*., 2016). These associations are then modeled in combination with future climate variables to predict shifts in allele frequencies, and thus the deviance between the genetic composition at contemporary time and the optimal composition in the future, termed ‘genetic offset’ (Fitzpatrick & Keller, 2015; Capblancq *et al*., 2020a). This approach has enabled research in long‐lived species (e.g. trees) for which field and glasshouse experiments are less feasible (Rellstab *et al*., 2016; Capblancq *et al*., 2020b). At its core however, genomic prediction of allele frequencies is essentially hypotheses, and few studies have validated whether predicted shifts coincide with observed allele frequency change (Lasky *et al*., 2023; Lind & Lotterhos, 2024).

Herbarium specimens can offer historical genetic data to validate genomic predictions to climate change (as briefly noted by Capblancq *et al*., 2020a; Rellstab *et al*., 2021). In essence, researchers could use contemporary associations between allele frequencies and climate to predict allele frequencies in historical climates (rather than future climates as per usual). These predictions can then be validated by sequencing historical populations through herbarium specimens and assessing the extent to which these models accurately hindcast allele frequency change. At this time, we could not find any studies that have yet validated genomic predictions using historical specimens. However, this approach would grant novel insight into the potential accuracy of future predictions, offering a powerful method of validation for the field of GEAs and shedding new light on the predictability of evolution more generally.

## Importance of digitization

As of 2023, only *c*. 21% of the Earth's herbarium specimens were digitized (Thiers, 2025). When it comes to the ability of these collections to supply genetic data, digitization is a critical step in increasing their accessibility and usefulness to researchers. Indeed, all the study designs discussed in this viewpoint require knowledge of the available specimens, their collection dates, and an accurate geolocation. Previous work has showcased how mass digitization could dramatically enhance the power and scope of our research – for example, the digitization of regional collections (e.g. all the specimens housed in Canadian institutions) could quintuple our ability to build statistical models to estimate geographic ranges (Eckert *et al*., 2024). Likewise, we expect that the complete digitization of Earth's remaining herbarium specimens would vastly increase the number of historical populations represented in the herbarium record, making it feasible to estimate population‐level genetic metrics across greater taxonomic, spatial, and temporal scales. That said, digitization alone is unlikely to alleviate the spatial, taxonomic, phylogenetic, and functional biases that have been identified in herbarium collections (Daru & Rodriguez, 2023; Eckert *et al*., 2024). These biases must be acknowledged in studies that use herbarium collections, including those generating genetic data, and contemporary efforts should be made to fill these gaps.

As the study of global change increasingly turns toward herbarium specimens as sources of morphological, functional, and genomic data – compiling these additional layers of data and metadata into standardized and digitally accessible *extended specimens* (Webster, 2018; Lendemer *et al*., 2020) will be critical for realizing the full potential of herbaria and their collections. The growing utility of these extended specimens to global change research has prompted calls for an open access global meta‐herbarium to enable data sharing and facilitate research (Davis, 2023). As individual studies employ herbarium specimens to investigate genetic change, researchers can contribute to these extended specimens by carefully standardizing and archiving the genetic data and metadata generated from each specimen. This step is critical for ensuring the utility of genetic data beyond its original study, including connecting genotypes to phenotypes that can be collected directly from digitized specimens, such as automated measures of phenology and morphology (Besnard *et al*., 2018; Ahlstrand *et al*., 2026). Generally, poor data and metadata archiving remain major obstacles in our understanding of global genetic variation (Pope *et al*., 2015; Toczydlowski *et al*., 2021) – as such, we point readers to Leigh *et al*. (2024) for best practices in genetic data archiving.

## Ethical considerations for working with herbarium specimens

While the mobilization of data from herbarium collections is and will continue to be transformative for biodiversity research, working with this data requires specific considerations. Globally, herbaria and their collections are a reflection of humanity's history of colonialism and conquest, such that the majority of specimens representing some of the most biodiverse ecosystems in the global south are often housed in institutions situated in the global north (Park *et al*., 2023). Acknowledging the colonial legacy still present in our collections is a critical first step towards a more inclusive and equitable global herbarium. We encourage researchers from the global north to consider these implications when conducting their research, and when possible, to consult those with local expertise on the focal plant populations and integrate their advice and knowledge into study designs. In addition to greater access to herbaria and their specimens, the global north also possesses greater access to genetic technologies and resources, and we encourage researchers to consider how the benefits of these resources can be shared equitably, in line with the Convention on Biological Diversity's Nagoya Protocol (Buck & Hamilton, 2011).

Additionally, herbarium collections represent a vast but limited resource, and the research discussed in this viewpoint requires destructive sampling of specimens (i.e. tissue sampling for DNA extraction). Given the colonial legacy of herbarium collections and the limited number of historical specimens, we implore researchers to consider how they can effectively and ethically use herbarium specimens and point readers to Davis *et al*. (2024) for best practices for both users and stewards. Recommendations include not relying on herbaria as a substitute for fieldwork, confirming specimen identification before sampling, avoiding sampling small collections and type specimens when possible, only sampling the amount of tissue needed for analysis, annotating the specimen after sampling, and archiving all generated data in open access repositories. This final step increases the utility of the data produced through destructive sampling and can prevent the need for further destructive sampling. As the value of herbaria and their collections continues to increase, the equitable and ethical use of herbarium specimens is necessary to preserve this resource for future generations.

## Conclusions

Humanity's persistent curiosity about the natural world has produced a vast trove of historical specimens, the true value of which was largely unbeknownst to the numerous collectors and naturalists who amassed the bulk of these collections. Now, these collections represent an invaluable source of biodiversity data. Indeed, herbarium specimens offer a unique opportunity to explore changes in historical genetic variation amid the ongoing era of global change (Meineke *et al*., 2018; Lang *et al*., 2019). Here, we highlight several exciting areas of research for which herbarium collections can offer unique insights, though the utility of genetic data obtained from herbarium specimens extends far beyond the applications discussed in this paper (Bieker & Martin, 2018; Lang *et al*., 2019; Burbano & Gutaker, 2023). As digitization continues and sequencing technologies and bioinformatic workflows improve, the genetic data housed in Earth's vast natural history collections will become increasingly accessible, unlocking the true potential of this invaluable resource.

## Competing interests

None declared.

## Author contributions

LE and IE conceived the idea for this study, which was refined with help from CPS and OR. LE developed the structure with help from all authors. LE wrote the introduction, and all authors wrote and edited the rest of the manuscript. LE made the figures. Supervision was provided by RDHB.

## Disclaimer

The New Phytologist Foundation remains neutral with regard to jurisdictional claims in maps and in any institutional affiliations.

## Data Availability

All data used in this article are publicly available and cited accordingly.
